# Environmental, maternal, and reproductive risk factors for childhood acute lymphoblastic leukemia in Egypt: a case-control study

**DOI:** 10.1186/s12885-016-2689-z

**Published:** 2016-08-20

**Authors:** Sameera Ezzat, Wafaa M. Rashed, Sherin Salem, M. Tevfik Dorak, Mai El-Daly, Mohamed Abdel-Hamid, Iman Sidhom, Alaa El-Hadad, Christopher Loffredo

**Affiliations:** 1 National Liver Institute, Menoufia University, Shibin El Kom, Egypt; 2 Children’s Cancer Hospital 57357, El Sayeda Zeinab, Cairo, 11441 Egypt; 3 National Cancer Institute, Cairo University, Cairo, Egypt; 4 School of Health Sciences, Liverpool Hope University, Liverpool, UK; 5 Faculty of Medicine, Minia University, Minia, Egypt; 6 Lombardi Cancer Center, Georgetown University, Washington, DC USA

**Keywords:** Acute Lymphoblastic Leukemia, Egypt, Cesarean Section, Ovulation induction

## Abstract

**Background:**

Acute lymphocytic leukemia (ALL) is the most common pediatric cancer. The exact cause is not known in most cases, but past epidemiological research has suggested a number of potential risk factors. This study evaluated associations between environmental and parental factors and the risk for ALL in Egyptian children to gain insight into risk factors in this developing country. Methods: We conducted a case-control design from May 2009 to February 2012. Cases were recruited from Children’s Cancer Hospital, Egypt (CCHE). Healthy controls were randomly selected from the general population to frequency-match the cumulative group of cases by sex, age groups (<1; 1 – 5; >5 – 10; >10 years) and region of residence (Cairo metropolitan region, Nile Delta region (North), and Upper Egypt (South)). Mothers provided answers to an administered questionnaire about their environmental exposures and health history including those of the father. Odds ratios (ORs) and 95 % confidence intervals (CI) were calculated using logistic regression with adjustment for covariates.

**Results:**

Two hundred ninety nine ALL cases and 351 population-based controls frequency-matched for age group, gender and location were recruited. The risk of ALL was increased with the mother’s use of medications for ovulation induction (OR_adj_ = 2.5, 95 % CI =1.2 –5.1) and to a lesser extend with her age (OR_adj_ = 1.8, 95 % CI = 1.1 – 2.8, for mothers ≥ 30 years old). Delivering the child by Cesarean section, was also associated with increased risk (OR_adj_ = 2.01, 95 % CI =1.24–2.81).

**Conclusions:**

In Egypt, the risk for childhood ALL appears to be associated with older maternal age, and certain maternal reproductive factors.

## Background

Worldwide, leukemia is consistently the most common type of childhood cancer in most countries [1–3], with the exception of some African countries in the Equator region where lymphoma is the most common type of childhood cancer [4]. Acute lymphoblastic leukemia (ALL) is the most prevalent subtype and represents 80 % of all childhood leukemia cases [5, 6]. The exact risk factors for ALL have yet to be identified, and most studies have yielded mixed results. Certain maternal and infant characteristics have been associated with the risk for ALL, For-example, increased maternal age was associated with increased risk of childhood cancer in US and UK [7, 8], but not in Iran [9]. Birth order may be an additional potential risk factor; several studies addressed birth order and its possible association with childhood ALL and have yielded inconsistent results [10–14]. Birth weight of the infant has also been found to be associated with an increased risk for ALL although the mechanism remains speculative. For every 500 g increase in birth weight, there was a 7 % increase in the risk of developing ALL [15]. A meta-analysis by Caughey and Michels confirmed this association between high birth weight and ALL [3]. Despite these consistent results, however, high birth weight accounts for only a small proportion of risk for leukemia [15]. Studies of the association of ALL with birth by Cesarean section yielded conflicting results; two studies found increased risk of ALL among children born by Cesarean section [16, 17], while others did not find any association [10, 18, 19]. Among the other risk factors studied is parental tobacco smoking; Lee et al. (2009) found that paternal smoking prior to conception was associated with elevated risk for ALL [20], possibly via genetic damage to sperm. The risk is even greater when the child is further exposed to the mother’s active or passive smoking in the postnatal period [21]. A recent study was done to assess the association of child’s and parents’ exposure to smoking with various phenotypic and molecular subtypes of childhood leukemia, and found that that the risk of childhood ALL was associated with history of paternal prenatal smoking combined with child’s postnatal passive smoking exposure. This risk was highest among B-cell precursor ALL with Tel/AML fusion gene [22].

Regarding pesticides exposure, two meta-analysis have found elevated risk of childhood leukemia with pesticides exposure during pregnancy [23, 24] . A pooled analysis from Childhood Leukemia International Consortium (CLIC) assessing parental pesticides exposures found that maternal occupational exposure to pesticides during pregnancy was not associated with increased risk of childhood ALL. However, paternal occupational exposure preconception to pesticides was associated with increased risk of ALL [25]. Another pooled analysis from CLIC, found that home pesticides exposure to mothers a few months before conception, during pregnancy and after pregnancy was associated with increased risk of childhood ALL [26]. In a recent meta-analysis by Chen et al, 2015 found that childhood exposure to indoor pesticides is associated with increased risk of leukemia [27].

The aim of the present study was to explore risk factors for childhood precursor B-cell ALL in an Egyptian population by examining potential environmental factors and parental health and reproductive factors that may contribute to the risk of this malignancy.

## Methods

We conducted a case-control study from May 2009 to February 2012. We contacted the parents of 317 eligible cases with a diagnosis of ALL, of whom 299 agreed to participate (94.3 %). Of the 371 population controls approached, the parents of 351 agreed to participate (94.6 %). The recruitment site for cases was the Children’s Cancer Hospital, Egypt (CCHE), which is a referral center for childhood malignancies. The CCHE serves the Cairo metropolitan and surrounding rural and semi-urban areas. Cases were eligible if they resided in Egypt, were diagnosed in the past 6 months with precursor B-cell (PBC) subtype immunophenotype of ALL, and were ≤14 years of age. Healthy controls were randomly selected from the general population to frequency-match the cumulative group of cases by sex, age groups (<1; 1 – 5; >5 – 10; >10 years) and region of residence (Cairo metropolitan region, Nile Delta region (North), and Upper Egypt (South)). From these regions, the number of required controls and their characteristics (age and sex) was determined by the sampling frame of the age and sex distributions of the cases; the sampling frame was updated every 6 months as cases were enrolled. The study recruiters visited a randomly selected district (urban neighborhood or rural village) within each region, and approached occupants of houses on a randomly chosen street. If none of the resident's children matched the required sex and age-group, the recruitment team moved to the next house. The recruitment team conducted the controls recruitment during weekends to make sure that the child and mother were at home. Trained interviewers approached the mothers of children, explained the purpose and procedures of the study, and obtained signed consents and assents from the mothers and any child ≥7 years of age, respectively. Interviewers administered the questionnaire face-to-face to mothers of cases and controls. The interview consisted of questions pertaining to the child, the mother and the father, asking about their socio-demographic characteristics, birth and medical conditions of the child, reproductive and medical history of the mothers (including child's birth order, number of siblings, history of miscarriage, child’s day care attendance, and mode of delivery). We also queried the occupations of the parents and environmental exposures of the parents such as smoking and pesticides, and use of hair dyes. While we were able to collect complete or near-complete data on most variables, birth weight data reported by the mothers were available only for 149 cases and 150 controls (the remainder was not able to recall this information).

### Statistical analysis

We used Student's *t*-test and Chi-squared test to compare continuous and categorical variables, respectively. For the latter tests, Fisher’s exact test was used when any expected cell count was <5. Using case-control status as the outcome variable, we used logistic regression to estimate the adjusted odds ratios (OR) and 95 % confidence intervals (95 % CI) of the associations between ALL and independent variables with adjustment for potential confounders as described below. All statistical tests were performed in Stata v.11 (Stata Corp, College Station, Texas, USA). All P values were two-tailed.

## Results

The distributions of socio-demographic characteristics of the study sample are presented in Table 1. The cases were 9 months older than the controls on average; since the number of controls recruited was more than the cases, this increase was mainly in the age groups 5 – 10 (28.8 % in cases versus 31.3 % in controls) and in the group who are older than 10 (9.0 % in cases versus 17.1 % in controls). The proportion of males was not significantly different between cases and controls, nor was urban versus rural residency, residing in Cairo versus other places, nor a family history of cancer (Table 1).

**Table 1:** Socio-demographic character istics of cases and controls

Socio-demographic characteristic	Cases (*n* = 299)	Controls (*n* = 351)	OR (95 % CI)^a^ *P* value
Age of the child			-
Mean (95 % CI) in months	61.0 (56.8 to 65.3)	70.2 (65.7 to 74.7)	0.004
Sex			
Male N (%)	178 (59.5)	203 (57.8)	1.07 (0.74 to 1.46) 0.69
Maternal urban/rural residence			
Rural residence N (%)	103 (34.4)	129 (36.7)	0.90 (0.65 to 1.25) 0.57
Location in Cairo vs elsewhere			
Cairo metropolitan area N (%)	123 (41.1)	139 (39.6)	1.07 (0.78 to 1.47) 0.77
Cancer in relatives			
Yes N (%)	55 (18.4)	60 (17.1)	1.08 (0.72 to 1.63) 0.89
Maternal age group			
≤ 22 years-N (%)	69 (23.1)	111 (31.6)	Reference
23-29 years-N (%)	153 (51.2)	165 (47.0)	1.49 (1.02 to 2.16)
30+ yr-N (%)	77 (25.7)	75 (21.4)	1.65 (1.06 to 2.55)
			P for trend: 0.02
Maternal Education level			
Secondary or higher education N (%)	226 (75.6)	211 (60.1)	2.05 (1.46 to 2.88) <0.001

^a^not adjusted

Maternal age at time of birth of the index child, as a continuous variable, was marginally associated with ALL risk (*P* = 0.08); however, when it was divided into categories (14–22, 23–29 and ≥ 30 year age groups), a linear increase in the unadjusted risk with increasing age became evident (age up to 22: referent; OR = 1.49 (95 % CI = 1.02–2.16) for age 23–29 years, and OR = 1.65 (95 % CI = 1.06–2.55) for age 30 year or above, P for trend: 0.02) (Table 1). The maternal age association remained unchanged after adjustment for child age and sex or maternal education level (OR_adj_ =1.76, 95 % CI = 1.12–2.76) for mothers >30 years old). The proportion of mothers with secondary or higher education was higher in cases than in controls (75.6 % vs 60.1 %; *P* < 0.001); the association between high educational level and ALL risk was statistically significant (OR = 2.05, 95 % CI = 1.46 – 2.88) (Table 1), even after adjustment for residence location (urban or rural, Cairo or elsewhere) and maternal age.

Associations between ALL and environmental and reproductive variables are shown in Table 2. Tobacco smoking was rarely reported by the mothers (1.3 % and 1.7 % among cases and controls, respectively). Father smoking during pregnancy of the mother in the index child was not associated with increased ALL risk (adjOR = 0.82, 95 % CI 0.60–1.20) (Table 2). Maternal exposure to ETS at work or at home from sources other than the husband was strongly associated with ALL risk (OR = 15.07, 95 % CI 6.11–40.55 after adjustment for child age and sex and maternal education). However, the amount of heavy smoke at room was more in the control than the cases 100.0 % and 48.1 % respectively (Table 2). For those who are exposed to the ETS, we have asked wither this exposure is at home, work or both, 100.0 % of the controls were at home versus 90.4 % of the cases at home, 7.7 % at work and 1.9 % both at home and work (data not shown). Neither the number of household smokers, paternal smoking behavior, or the child's exposure to cigarette smoke at home were significantly associated with ALL risk. No statistically significant associations were observed for hair dyes. Very few mothers reported exposure to agriculture pesticides (3 % and 1.4 % of the cases and controls respectively). Moreover, there was no significant difference between cases and controls regarding the mother’s use of home insecticides (data not shown).

**Table 2:** Associations between environmental, maternal, and reproductive factors and ALL risk among Egyptian children

Variable	Cases *n* = 299	Controls *n* = 351	OR (95 % CI)	Adjusted OR^a^ (95 % CI)	Adjusted OR^b^ (95 % CI)
Father cigarette smoking during pregnancy period N (%)	138 (46.2)	185* (53.2)	0.75 (0.55-1.03)	0.75 (0.55-1.03)	0.82 (0.60-1.12)
Maternal exposure to environmental tobacco smoke from sources other than the husband N (%)	52 (17.4)	5 (1.4)	14.6 (5.74 to 37.0)	14.26 (5.59 to 36.30)	15.07 (6.11 to 40.55)
Amount of smoke in the room (for those reported exposure to ETS). (heavy versus Light) N (%)	25/52 (48.1)	5/5 (100.0)	1.20 (1.02-1.40)	NA	NA
Hair dye use N (%)	56 (18.7)	51 (14.5)	1.44 (0.95 to 2.19)	1.36 (0.89 to 2.07)	1.25 (0.81 to 1.92)
Number of pregnancies (Mean)	3.2	3.5	0.91 (0.84 to 1.00)	0.94 (0.86 to 1.03)	0.98 (0.89 to 1.07)
Number of deliveries N (%)					
1	39 (13.0)	33 (9.4)	Reference	Reference	Reference
2	114(38.1)	116 (33.0)	0.83 (0.48 to 1.40)	0.93 (0.54 to 1.60)	1.15 (0.65 to 2.03)
3	86 (28.7)	115 (32.7)	0.63 (0.36 to 1.08)	0.74 (0.42 to 1.30)	0.99 (0.55 to 1.78)
4+	60 (20.0)	87 (24.7)	0.58 (0.33 to 1.03)	0.73 (0.40 to 1.35)	1.36 (0.70 to 2.62)
Number of siblings (Mean)	2.6	2.9	0.80 (0.70 to 0.92)	0.91 (0.81 to 1.03)	0.97 (0.86 to 1.09)
Birth order (Mean)	2.2	2.3	0.90 (0.80 to 1.01)	0.84 (0.73 to 0.96)	0.89 (0.77 to 1.02)
Cesarean section N (%)	121 (40.4)	89 (23.3)	2.01 (1.44 to 2.81)	1.90 (1.35 to 2.66)	1.68 (1.18 to 2.38)
Non-term birth N (%)	54 (18.0)	7 (1.9)	10.80 (4.85 to 24.2)	10.61 (4.73 to 23.7)	10.66 (4.72 to 24.1)
Induction of Ovulation N (%)	26 (8.6)	13 (3.7)	2.47 (1.25 to 4.9)	2.53 (1.27 to 5.05)	2.51 (1.24 to 5.05)

^**a**^adjusted for child age and sex

^**b**^adjusted for child age and sex, and maternal educational level

*2 controls missing data

*NA* Not Applicable

We observed differences between cases and controls in Cesarean section and non-term birth (Table 2). None were differentially associated with age, sex or education level. We tested the independence of these variables in a multivariable model and in the presence of others, they remained statistically significant (*P* ≤ 0.001). Treatment for induction of ovulation also showed an association that remained significant after adjustment for age, gender and education (Table 2).

The number of deliveries, a categorical variable (1, 2, 3, and 4+) was associated with decreased risk in childhood ALL; compared to one delivery as the reference, the ORs were 0.83, 0.63 and 0.58 for 2, 3 and 4 + deliveries, respectively (*P for trend = 0.02*). Likewise, the number of siblings also showed an inverse correlation with risk. However, adjustment for maternal education level attenuated the associations of number of deliveries and the number of siblings, and they became non-significant.

Birth order showed an inverse association with childhood ALL risk, but was not statistically significant. It became significant after adjusting for age and sex so we performed a stratified analysis for males and females separately. Birth order and ALL risk was observed in males only (OR = 0.84, 95 % CI = 0.71 – 0.99) but was not statistically significant after adjustment for maternal educational level.

Birth weight data were available for 149 cases and 150 controls (46 % of the total sample). To rule out any selection bias, we compared the cases and controls in this subset for sociodemographic characteristics, and found no difference between the subsamples with and without birth weight data. To validate the representativeness of this subset, we re-examined the associations described above in this subset. All but two associations (number of children and maternal age) remained statistically significant, and all ORs remained in the same direction as in the original analysis (data not shown). In the subjects (*n* = 299) with birth weight data, the mean birth weight (cases and controls combined) was 3190.6 g (SEM = 48.8 g (SD: 809.6 g); 95 % CI = 3120.4 – 3312.3). The distribution was not normal and therefore we used non- parametric methods of analysis. The median birth weight was 3000 g (interquartile range: 2750 – 3500 g), which was also the mode. In case-control comparisons, there was a marginally significant difference in birth weight medians between cases (3,200 g) and controls (3,000 g) (P (median test) = 0.06). We did not collect data on gestational age, but we did ask whether the child was born on the expected date or not. Restricting the analysis to those born at expected date (term) yielded a *P* value of 0.01 for the association between ALL and birth weight. The association was not confounded by any of the variables from the questionnaire. The examination of a potential sex effect on the birth weight association revealed male-specificity: more male cases than male controls had birth weight above the median value (58.0 vs 35.0 %; OR = 2.56), but not in females (47.5 vs 48.5 %). The difference in birth weight association between males and females also reached statistical significance (P for interaction with sex = 0.04).

## Discussion

In this case-control study of childhood ALL in Egypt, we observed statistically significant associations with the mother’s age, high educational attainment, and some reproductive factors, i.e., ovulation induction and non-term birth.

Our finding about increased risk of ALL with increasing maternal age is consistent with a pooled analysis of population based data in the United States [7], and with an earlier study conducted in UK for ALL cases from England and Wales [8]. However, our results contradict the findings from an Iranian study, which reported no statistically significant difference of the mother’s age at time of childbirth between the case and control groups [9]. Although the authors explained this observation as possibly due to younger age of pregnancy in rural areas in Iran and other associated genetic and environmental factors, we observed no such association in our study. Although paternal age was correlated with maternal age, it was not associated with the risk of ALL and it did not confound the association of the significant risk factors when included in the model.

Maternal high educational level was previously reported to be a risk factor in studies from Greece [28], the Netherlands [29], and the USA [16, 19]. Education level is usually considered a proxy for socioeconomic level [28], and other studies have shown a link between childhood leukemia and higher socioeconomic status using the same or different social class indicators, although the associations were not always in the same direction [30–32]. Even in developed countries, higher socioeconomic status was found to be associated with childhood leukemia risk [33, 34]. The association of maternal education level with childhood ALL risk in Egypt might also reflect an association with western life style. A systematic review done by Adam el al, 2008, concluded that most of the studies done on socioeconomic status (SES) are heterogeneous and there is no evidence to support the relation between SES and childhood leukemia, Moreover, they concluded that the association found in some studies might be related to selection bias [32]. Maternal high education level associated with increased risk of ALL in our study could be a true association or represent selection bias which cannot be ruled out completely. However, we have a high participation rate from cases and controls (94.3 and 94.6 % respectively), and we have matched controls to cases on region and urban/rural place of residence to control for socioeconomic factors. Moreover father education didn’t differ between cases and controls, and we have adjusted for mother education in all other exposures.

Despite the fact that maternal environmental tobacco smoke exposure during pregnancy at work or at home from sources other than the husband was the strongest association with childhood ALL status in our study, neither paternal smoking nor the child's exposure to cigarette smoke showed statistically significant associations. These differences may suggest the possibility of a critical window of environmental exposure in the prenatal exposure period, consistent with some models of the pathogenesis of ALL [35, 36]. Measurement error is a problem in epidemiological studies demanding self-reports. Recall bias, where differential misclassification might have affected the results of the ETS, where mothers of the cases over reported exposure to ETS and/or mother of controls underreported this exposure. Therefore, the results of ETS should be taken cautiously under validated in a larger study and assessing with biomarkers of exposures.

The associations we observed regarding the number of pregnancies/deliveries and birth order appeared similar to what has been reported before [8, 9, 12], but lost statistical significance after adjustment for maternal education level. Birth order is one of the most studied associations in childhood ALL [37], and it is important to verify its independence in future studies. Interestingly, similar to our results, in a comprehensive study of birth order in non-Hodgkin lymphoma, adjustment for socioeconomic status removed the statistical significance of birth order association, and suggested that selection bias related to socioeconomic status may be responsible for the association [38].

We observed an association between ALL risk and ovulation induction treatment. Previous studies reported positive associations with different types of fertility treatment and ALL risk [39–41]. A study in France showed an association between leukemia and ovulation induction but not with in vitro fertilization [42]. Another study by the same group in France did not confirm this association nor with specific types of ovulation induction medication [43]. Finally, it is possible that the association with ovulation induction treatment might be related to factors underlying fertility problems [44], and not the treatment itself.

The association of Cesarean section with ALL risk in our study has been reported before and is biologically plausible, since it is possible that Cesarean birth may affect the developing immune system through several mechanisms, including exposure to organisms that affect the immune response across the life span [45], and alteration of methylation patterns [46]. Recall bias is unlikely to have been an impact on risk factors such as induction of ovulations medications or CS delivery, since there was no public perception about the risk of these two exposures and they are not subjective exposures.

There was a positive association in our study of birth weight with ALL risk, but this analysis had to be restricted to a subset of the sample, and thus should be interpreted with caution. Nevertheless, despite the small sample size and our reliance on maternally reported data, the results were similar to those reported by Dorak et al. from northern England [47], who also suggested that the sexual dimorphism has biological plausibility [48].

The present study had several limitations. By virtue of its case-control design, we had to rely on questionnaires to retrospectively obtain environmental exposure data rather than biomarkers (e.g. tobacco smoke exposure was not assessed by blood cotinine levels). Future studies should test hypotheses generated in the present study using a prospective design to address the issues of recall bias and recall errors.

## Conclusions

To our knowledge, the present study is the most comprehensive and largest study of environmental and maternal risk factors of childhood ALL in a developing country. Studies in such countries, which harbor 90 % of the world's children, are desirable not only to identify unique risk factors, but also to shed light on certain environmental risk factors that may no longer be common enough to study in developed countries. Such clues will help to better identify biological mechanisms in ALL development and pave the way to prevention. Our finding regarding ETS should be considered cautiously since it might represent recall bias until validated in another study.

## Abbreviations

ALL: Acute Lymphoblastic Leukemia
CCHE: Children’s Cancer Hospital, Egypt
IRB: Institutional Review Board
OR: Odds Ratio
